# Keypoint detection network for needle localization on intra-procedural MRI in MRI-guided liver interventions

**DOI:** 10.1007/s11548-026-03592-5

**Published:** 2026-03-30

**Authors:** Wenqi Zhou, Qing Dai, Omar Curiel, Tsu-Chin Tsao, Jason Chiang, David S. Lu, Holden H. Wu

**Affiliations:** 1 Department of Radiological Sciences, University of California Los Angeles, 300 UCLA Medical Plaza, Suite B119, Los Angeles, CA 90095 USA; 2 Department of Bioengineering, University of California Los Angeles, Los Angeles, CA USA; 3 Department of Mechanical and Aerospace Engineering, University of California Los Angeles, Los Angeles, CA USA

**Keywords:** Interventional MRI, Real-time MRI, Interventional needle localization and tracking, Keypoint localization, Segmentation

## Abstract

**Purpose:**

Segmentation neural networks have demonstrated promising results for interventional needle localization on MRI. However, these networks require large training datasets with tedious annotation processes and additional post-processing steps that may introduce variability in the final localization results. This study aimed to develop keypoint detection networks for direct localization of the needle entry point and tip on intra-procedural liver MRI with a more efficient annotation process and more robust performance.

**Methods:**

2D and 3D keypoint detection networks were developed by enhancing the stacked hourglass model and incorporating multi-task learning of predicting part affinity fields that connect the keypoints. The proposed networks were evaluated on intra-procedural single-slice controlled-breathing (SS-CB) and multislice controlled-breathing (MS-CB) images acquired from pre-clinical MRI-guided percutaneous liver intervention in thirteen in vivo pig subjects and compared with the results of segmentation-based UNet and Swin Transformer networks and human intra-reader variation.

**Results:**

The 2D and 3D keypoint detection networks achieved median needle tip and axis localization errors of 1.56 mm (1 pixel) and 1.1° for the SS-CB datasets, and 2.21 mm (~ 1.5 pixel) and 1.45° for the MS-CB datasets, respectively. Average computational times were 10 ms (2D) and 30 ms (3D). The needle localization accuracy of the keypoint networks was significantly (Wilcoxon signed-rank tests *p* < 0.001) higher than the UNet and Swin Transformer segmentation-based results and comparable to human intra-reader variation.

**Conclusion:**

The proposed keypoint detection networks achieved rapid pixel-level needle localization on single-slice and multislice intra-procedural liver MRI with higher accuracy and a more efficient annotation process compared to segmentation-based models.

**Supplementary Information:**

The online version contains supplementary material available at https://doi.org/10.1007/s11548-026-03592-5.

## Introduction

Image-guided percutaneous interventions, such as needle-based targeted biopsy and ablation, are promising strategies for liver cancer diagnosis and treatment [1, 2]. Magnetic resonance imaging (MRI) provides excellent soft-tissue contrast to depict different types of liver tumors, such as hepatocellular carcinoma and colorectal liver metastases, which can be difficult to visualize intra-procedurally on computed tomography (CT) or ultrasound (US) [3]. MRI also offers advantages of non-ionizing radiation, flexible scan plane orientations, and capability for continuous guidance in real-time [4], making it an emerging option for guiding needle-based percutaneous liver interventions in clinical settings [5].

Current MRI-guided freehand needle-based liver interventions generally follow the step-and-shoot workflow, which includes iterations of in-bore intra-procedural MRI and out-of-bore needle manipulation [6]. In each iteration, time-efficient and accurate needle localization on intra-procedural MRI is critical for guiding out-of-bore needle manipulation. However, manual needle localization by the interventionalist requires interpretation and adjustment of multiple images with expert awareness of the anatomy, imaging orientations, needle trajectory, and target lesions, which adds cognitive burden over the iterative workflow steps and prolongs the procedural time [7, 8].

To assist the interventionalist’s decision-making, computer-aided methods have been proposed to automate the process of needle localization [9]. Among these automatic methods, neural networks that segment the needle feature and localize the needle tip and entry point based on the segmentation masks have been developed for 2D and 3D intra-procedural MRI and achieved pixel-level accuracy within tens to hundreds of milliseconds [10–15]. However, these methods share a limitation of requiring a large training dataset with segmentation masks that are time-consuming to annotate. In addition, post-processing is needed to extract the needle tip and entry point from the segmentation masks. Uncertainty in the segmentation boundaries (e.g., due to misalignment of the needle and the imaging plane) can lead to variability during post-processing and affect the accuracy of final localization.

To localize the interventional needle on MRI, fully segmenting the needle feature may be unnecessary since only the coordinates of the needle tip and entry point are required. Therefore, keypoint detection networks are inherently suited for this task. Keypoint detection networks have achieved promising performance on natural images [16, 17], and have also been employed for interventional needle tracking in US images, motivated by the difficulty of defining accurate segmentation masks of the needle shaft [18–20]. Similar challenges exist in localizing the needle on intra-procedural MRI, especially when the needle is misaligned with the imaging plane, but keypoint detection networks have not yet been explored for this task.

Deep learning-based needle localization in intra-procedural MRI is currently limited by the need for robust performance across varying sequence types and parameters used in the workflow [21]. For MRI-guided liver interventions, 2D breath-hold imaging is often employed to suppress motion [22], but repeated breath-holds can prolong the procedures due to the time for instructions and breaks, and may fail for patients unable to perform breath-holds [23]. Alternatively, 2D images acquired during free-breathing or ventilator-controlled breathing [24] can be used, albeit often with compromises in spatial resolution or coverage [25]. Furthermore, to capture the whole needle feature, 2D imaging planes are prescribed along the needle trajectory [15], yet misalignment from breathing motion or tissue interaction still occur [11, 12]. To avoid frequent imaging plane adjustments, 2D multislice acquisitions—usually three parallel slices—are preferred over single-slice imaging [26]. Therefore, automatic needle localization methods that provide robust performance across these different types of MR images are required.

The main objective of this study is to develop and evaluate keypoint detection networks for needle localization on pre-clinical intra-procedural liver MRI. We first developed a 2D keypoint detection network for needle localization on 2D single-slice breath-hold and controlled-breathing MRI. Then we extended the model to 3D for 2D multislice (3-parallel-slice) breath-hold and controlled-breathing MRI. We compared the performance of the proposed networks to results from 2D and 3D segmentation networks and human annotations.

## Methods

### MRI-guided interventional experiments in pigs

**Fig. 1:**
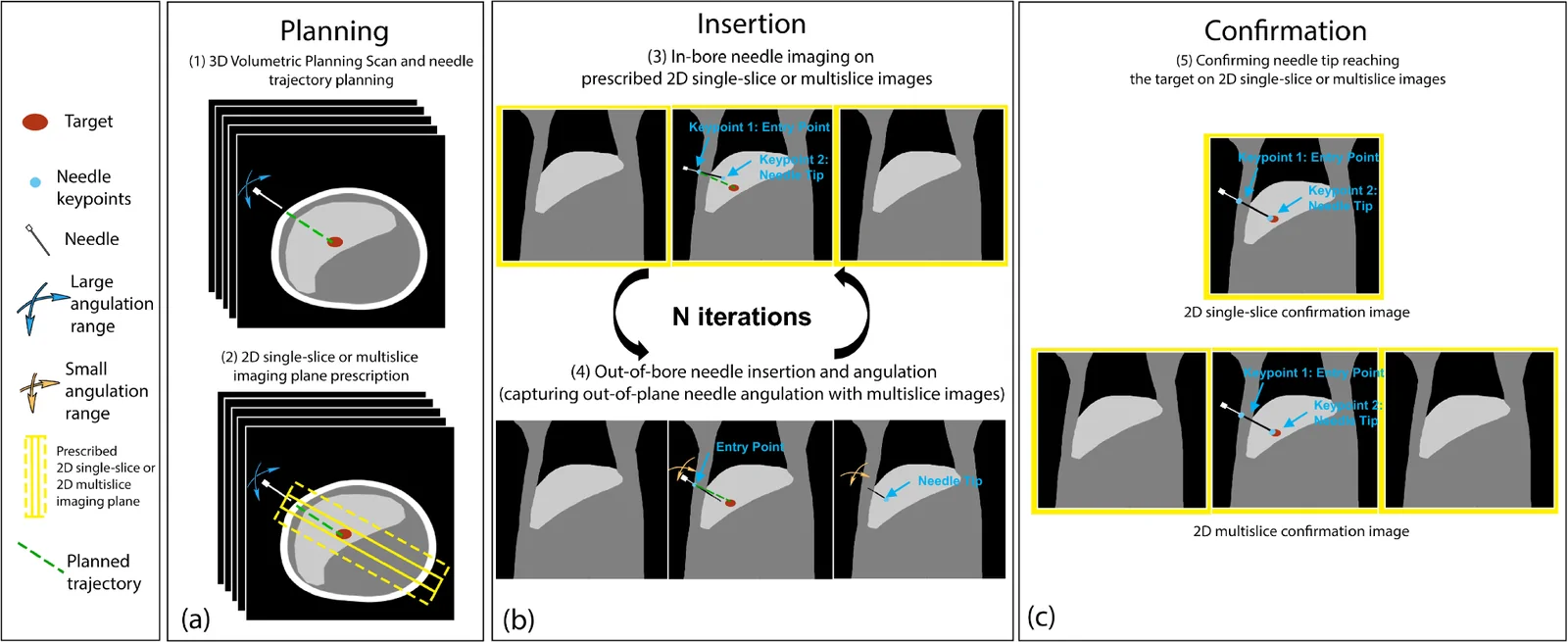
Freehand step-and-shoot MRI-guided percutaneous liver intervention workflow. In the planning stage **a**, 3D MRI were used to identify the target and to plan the needle insertion trajectory (1). Next, 2D single-slice or multislice images were prescribed along the planned needle trajectory (2). In the insertion stage **b**, prescribed 2D single-slice and/or multislice images were acquired to monitor needle advancement. The multislice images enabled monitoring of small out-of-plane needle angulation. In the confirmation stage **c**, 2D single-slice and/or multislice images were acquired to confirm the needle tip reached the target

**Fig. 2:**
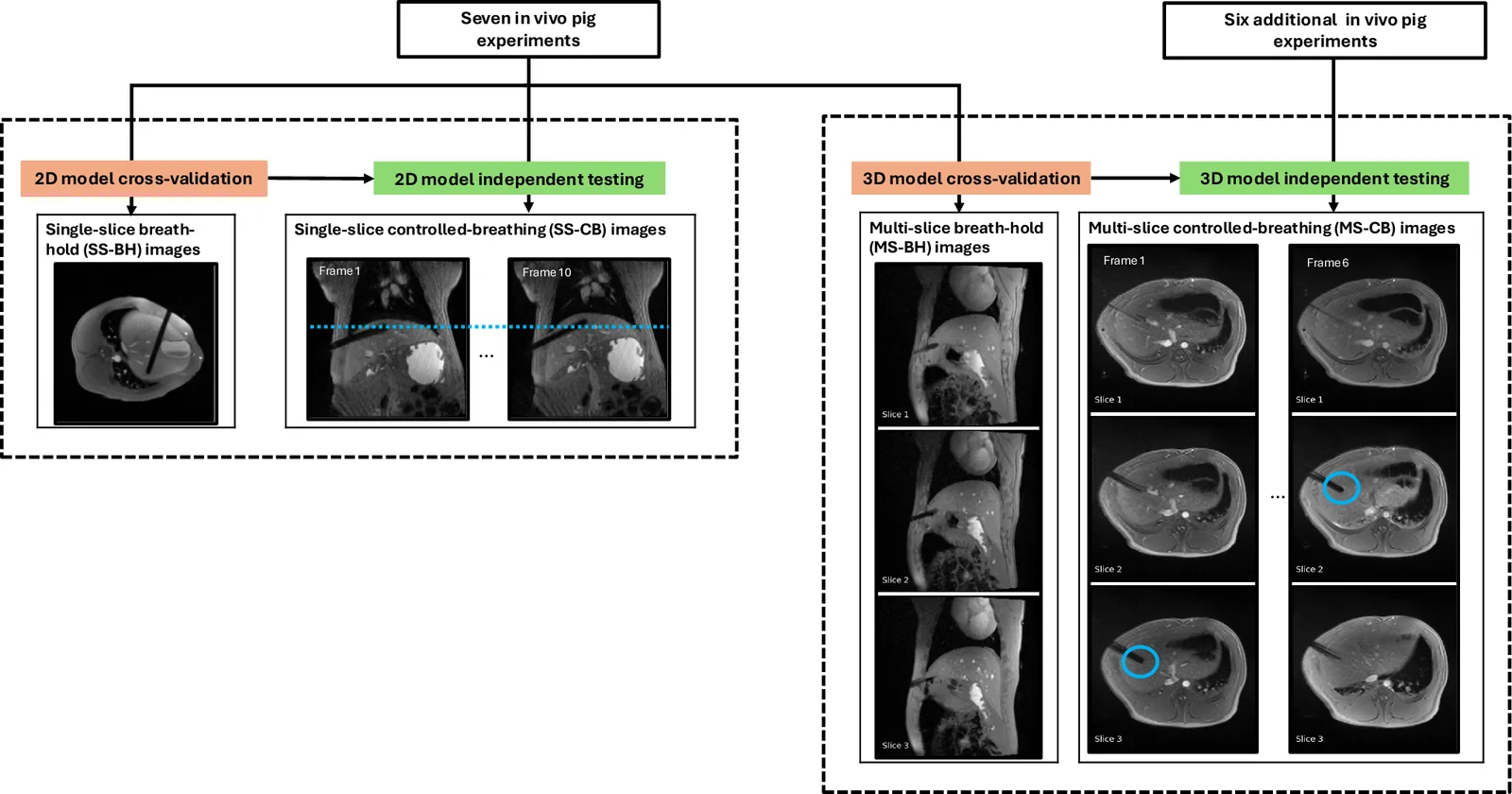
Diagram of the training strategies and the four MRI datasets used in this study. The 2D and 3D keypoint detection models were trained and cross-validated with the single-slice and multislice breath-holding images respectively and tested with single-slice and multislice controlled-breathing images

In an animal research committee-approved study, we performed MRI-guided liver interventions in thirteen healthy female pigs (32–36 kg; Yorkshire, Premier Biosource, Ramona, CA). Target lesions were first created in the liver using radiofrequency or microwave ablation under US guidance, followed by MRI-guided needle placement at the target lesions on a 3T scanner (MAGNETOM Prisma, Siemens, Erlangen, Germany) performed by an experienced interventional radiologist (over 20 years of experience) based on the freehand step-and-shoot workflow in Fig. 1. The pigs were under anesthesia and breathing was controlled by a ventilator during the procedures. In the insertion and confirmation stages, oblique axial and oblique sagittal 2D single-slice images and 2D multislice (3-parallel-slice) images with the imaging plane aligned with the planned needle trajectory were acquired during breath-hold and ventilator-controlled-breathing for model training and evaluation as shown in Fig. 2.

### Intra-procedural MRI datasets

Intra-procedural 2D single-slice breath-hold (SS-BH) and single-slice controlled-breathing (SS-CB) images were collected using a golden-angle (GA) ordered radial spoiled gradient-echo (GRE) sequence [12] during 7 experiments with parameters shown in Table 1. The SS-BH images were reconstructed using a non-Cartesian conjugate gradient sensitivity encoding (CG-SENSE) approach [27] with a temporal footprint of 200 radial spokes per image and temporal resolution of 0.76–1.26 s. The SS-CB image series were reconstructed using a sliding-window CG-SENSE approach with a temporal footprint of 70 radial spokes per image (266–441 ms) and temporal resolution of 60–100 ms (sliding-window update every 16 radial spokes).

**Table 1:** 2D single-slice (SS) MRI datasets and imaging parameters

	2D SS-BH GA radial GRE	2D SS-CB GA radial GRE
TR/TE (ms)	3.8/1.72; 5.08/3; 6.3/2.85	3.8/1.72; 5.08/3; 6.3/2.85
Field-of-view	300 × 300 mm^2^	300 × 300 mm^2^
Number of slices	1	1
In-plane resolution	1.56 × 1.56 mm^2^	1.56 × 1.56 mm^2^
In-plane matrix size	192 × 192	192 × 192
Slice thickness	5 mm	5 mm
Flip angle	9°	9°
Parallel Imaging Factor	1.5 (CG-SENSE)	4.3 (CG-SENSE)
Temporal Footprint	0.76–1.26 s (200 radial spokes) per image	266–441 ms (70 radial spokes) per image
Temporal Resolution	0.76–1.26 s (breath-holding)	60–100 ms (sliding window update by 16 spokes) (controlled-breathing)
Size of dataset	490 images from seven in vivo experiments	560 images (10 real-time image series) from seven in vivo experiments
Range of insertion depth/angle in image coordinates	25–110 mm − 178° to 180°	27–79 mm − 45.0° to 32.59°
Imaging plane orientation	Oblique axial, oblique sagittal, and oblique coronal; aligned with the planned needle trajectory	
Annotation	Keypoints (needle tip and entry point); Segmentation masks	Keypoints (needle tip and entry point)

The SS breath-hold (SS-BH) and SS controlled-breathing (SS-CB) images were acquired with the same golden-angle (GA) ordered radial gradient-echo (GRE) sequence at 3 T. *TR* Repetition time, *TE* Echo time, *CG-SENSE* Conjugate gradient sensitivity encoding

Intra-procedural 2D multislice breath-hold (MS-BH) images were collected during 7 experiments using the same sequence and reconstruction method as the SS-BH images with temporal resolution of 2.28–3.78 s per image set (3 slices, 200 radial spokes/slice). Multislice controlled-breathing (MS-CB) images were collected during 6 additional experiments using a Cartesian GRE sequence and reconstructed with generalized autocalibrating partially parallel acquisitions (GRAPPA) [28] with a temporal resolution of 1.8 s per image set. The scanning parameters of MS-BH and MS-CB images are reported in Table 2.

**Table 2:** 2D multislice (MS) MRI datasets and imaging parameters

	2D MS-BH GA radial GRE	2D MS-CB Cartesian GRE
TR/TE (ms)	3.8/1.72; 5.08/3; 6.3/2.85	5.53/2.6
Field-of-view	300 × 300 mm^2^	300 × 300 mm^2^
Number of slices	3	3
In-plane resolution	1.56 × 1.56 mm^2^	1.56 × 1.56 mm^2^
In-plane matrix size	192 × 192	192 × 192
Slice thickness	5 mm	5 mm
Flip angle	9°	12°
Parallel Imaging Factor	1.5 (CG-SENSE)	1.8 (GRAPPA)
Temporal Resolution	2.28–3.78 s per image set (breath-holding)	1.8 s per image set (controlled-breathing)
Size of dataset	210 sets of multislice images from seven in vivo experiments	340 sets of multislice images (20 real-time image series) from six additional in vivo experiments
Range of insertion depth/angle in image coordinates	28–81 mm − 179° to 179°	11–54 mm − 29.22° to 116.51°
Imaging plane orientation	Oblique axial, oblique sagittal, and oblique coronal; aligned with the planned needle trajectory	
Annotation	Keypoints (needle tip and entry point); Segmentation masks	Keypoints (needle tip and entry point)

The MS breath-hold (MS-BH) images were acquired with the golden-angle (GA) ordered radial gradient-echo (GRE) sequence, and MS controlled-breathing (MS-CB) images were acquired with the Cartesian GRE sequence at 3 T. *TR* Repetition time, *TE* Echo time, *CG-SENSE*: Conjugate gradient sensitivity encoding, *GRAPPA* GeneRalized Autocalibrating Partial Parallel Acquisition

### Reference needle annotation on intra-procedural MRI

The needle tip and entry point were defined as the keypoints of interest (Fig. 1). The slice index of the needle tip and entry point was regarded as the third dimension of the keypoint coordinates. Under the supervision of the interventional radiologist, a trained researcher manually annotated the needle tip and entry point locations (i.e., marked two points) in the four MRI datasets. The annotations were performed twice, with a washout period of two weeks in between, to assess human intra-reader variation. Reference heatmaps were created based on the coordinates of needle tip and entry points following the method used by the original stacked hourglass model [16]. Needle feature masks were also annotated for the SS-BH and MS-BH datasets for training segmentation-based models.

### 2D and 3D needle keypoint detection models

**Fig. 3:**
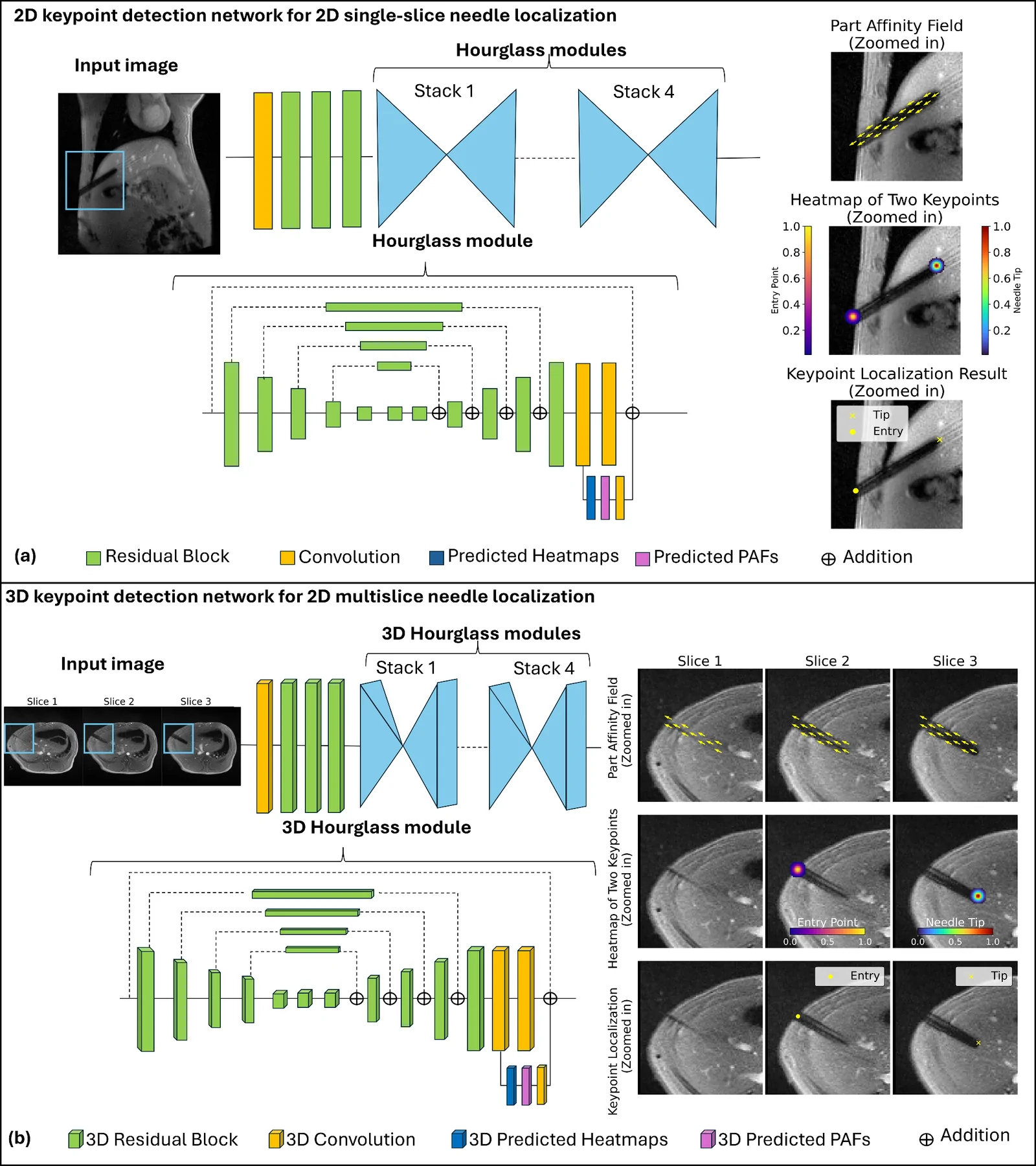
Structure of **a** 2D and **b** 3D keypoint detection networks. Four cascaded hourglass modules were used for the proposed networks. The networks predicted two heatmaps for needle tip and needle entry points, respectively, along with the Part Affinity Fields (PAFs) representing the connectivity of the two points. For visualization, the heatmaps of the two classes (tip, entry point) were overlaid on one input image with different colormaps, showing only regions with prediction scores above zero

We developed a 2D keypoint detection model for needle localization based on the 2D stacked hourglass model structure (Fig. 3). Besides predicting the heatmap, we enhanced the robustness of the model by predicting Part Affinity Fields (PAFs) [29], which encode the directional vector field between the needle tip and the entry point. The PAFs were defined by Eq. (1), where *p* refers to a point in the image. Our proposed model performed multi-task learning of both the heatmaps and PAFs using a combined weighted mean squared error (MSE) loss for the heatmaps and PAFs (Eq. 2). A 3D keypoint detection model that can capture the cross-slice information on multislice inputs was also developed by extending the 2D model structure to 3D (Fig. 3). The 3D keypoint localization network outputs 3D heatmaps and 3D PAFs as multitask learning outputs. Predicted keypoint coordinates were obtained by applying non-maximum suppression to the output heatmaps [16].

1 $$ {\mathrm{PAF}}({\mathrm{p}}) = \left\{ {\begin{array}{*{20}c}    {\frac{{p_{{entry}}  - p_{{tip}} }}{{\parallel p_{{entry}}  - p_{{tip}} \parallel _{2} }}} & {if\frac{{\parallel (p_{{entry}}  - p_{{tip}} ) \times (p_{{entry}}  - p)\parallel _{2} }}{{\parallel p_{{entry}}  - p_{{tip}} \parallel _{2} }} < 5}  \\    0 & {otherwise}  \\   \end{array} } \right., $$

$$ \begin {aligned}&{\text{Weighted MSE}}\left( {heatmap} \right)\\  &\quad = \frac{1}{{\sum _{i} w_{i} }}\sum _{i} w_{i}  \cdot \parallel heatmap_{i}  - \widehat{{heatmap_{i} }}\parallel _{2} , \\w_{i} & = \left\{ {\begin{array}{*{20}c}    3 & {if{\text{ }}heatmap_{i}  > 0}  \\    1 & {otherwise}  \\   \end{array} } \right.,\end {aligned} $$

$$ {\text{Weighted MSE}}\left( {PAF} \right) = \frac{1}{{\sum _{i} w_{i} }}\sum _{i} w_{i}  \cdot \parallel PAF_{i}  - \widehat{{PAF_{i} }}\parallel _{2} ,w_{i}  = \left\{ {\begin{array}{*{20}c}    3 & {if\;PAF_{i} > 0}  \\    1 & {otherwise}  \\   \end{array} } \right., $$

2 $$ Loss = \lambda _{1} Weighted\;MSE\left( {heatmap} \right) + \lambda _{2} Weighted\;MSE\left( {PAF} \right),\;\lambda _{1}  = 0.6,\;\lambda _{1}  = 0.4 $$

The 2D and 3D keypoint detection networks were trained with the parameters in Table 3. We performed seven-fold cross-validation of the 2D keypoint detection network on the SS-BH dataset, and then independently tested it on the SS-CB dataset. The 3D keypoint network was first pretrained on a synthetic dataset of 888 images (example in Supplemental Fig. S1) with needle features simulated using Fourier-based off-resonance artifact simulation (FORECAST) [12] and overlaid on 3-parallel-slice images extracted from 3D gradient-echo MRI collected during the pig experiments (TR/TE: 3.91/1.23 ms, resolution: 1.35 × 1.35 mm^2^, slice thickness: 1.5 mm) [30]. We then trained and cross-validated the pre-trained 3D keypoint detection network with the MS-BH dataset from 7 experiments. The trained 3D keypoint model was evaluated on an independent testing set of MS-CB images acquired from 6 additional experiments (Fig. 2).

**Table 3:** Model training parameters and training strategies

	2D keypoint network	2D Swin Transformer	2D UNet	3D keypoint network	Per-slice Swin Transformer	3D UNet
Hardware	NVIDIA RTX A6000 GPU card (48 GB memory)					
Loss function	Weighted MSE loss	Dice loss	Dice loss	Weighted MSE loss	Dice loss	Dice loss
Learning rate	0.001			Pre-train: 0.01; Fine-tune: 0.001		
Optimizer	Adam			Adam		
Batch size	4			2		
Number of epochs	500			Pre-train: 500; Fine-tune:300		
Trainable weights	823,408	48,962,404	1,625,161	1,893,108	48,962,404	2,549,929
Training time	~ 6 h	~ 7 h	~ 6 h	~ 14 h	~ 20 h	~ 14 h
Inference time	~ 10 ms	~ 12 ms	~ 10 ms	~ 30 ms	~ 35 ms	~ 30 ms
Cross validation	sevenfold cross-validation on SS-BH dataset			sevenfold cross-validation on MS-BH dataset		
Independent testing	SS-CB dataset from seven experiments			MS-CB dataset from six additional experiments		
Data augmentation	On-the-fly data augmentation including random rotation, flipping, translation, zooming, and additive Gaussian noise					

*MSE* Mean squared error, *SS* Single-slice, *MS* Multislice, *BH* Breath-hold, *CB* Controlled-breathing

### 2D and 3D needle segmentation models

For comparison, we trained and evaluated 2D UNet and 2D Swin Transformer for the SS-CB dataset, and 3D UNet and per-slice 2D Swin Transformer for the MS-CB dataset. Training details are provided in Table 3. For the per-slice 2D Swin Transformer, the model was trained on single slices extracted from multi-slice images and applied independently to each slice during evaluation. Needle geometry was extracted from the single-slice or combined 3-slice needle feature segmentation masks through orthogonal distance regression to determine the needle axis, with needle tip and entry points determined at axis-mask intersections [11, 13].

### Performance evaluation metrics

The Euclidean distance between the predicted needle tip and the reference in mm (${{\boldsymbol{\varepsilon}}}_{{\boldsymbol{t}}{\boldsymbol{i}}{\boldsymbol{p}}}$), the Euclidean distance between the predicted needle entry point and the reference in mm (${{\boldsymbol{\varepsilon}}}_{{\boldsymbol{e}}{\boldsymbol{n}}{\boldsymbol{t}}{\boldsymbol{r}}{\boldsymbol{y}}}$), and the angle between the predicted needle axis and the reference in degrees ($\boldsymbol{\alpha }$) were calculated. For the multislice datasets, ${{\boldsymbol{\varepsilon}}}_{{\boldsymbol{t}}{\boldsymbol{i}}{\boldsymbol{p}}}$, ${{\boldsymbol{\varepsilon}}}_{{\boldsymbol{e}}{\boldsymbol{n}}{\boldsymbol{t}}{\boldsymbol{r}}{\boldsymbol{y}}}$, and $\boldsymbol{\alpha }$ were calculated in 3D space. We also calculated the number and percentage of outliers, where needle tip outliers were defined as ${{\boldsymbol{\varepsilon}}}_{{\boldsymbol{t}}{\boldsymbol{i}}{\boldsymbol{p}}}$ >5 mm, as liver lesion diameters of 5–10 mm or larger are typically considered as clinically relevant sizes for intervention [31]. For consistency, the same 5-mm threshold was applied to characterize outliers for needle entry point localization.

### Statistical analysis

We compared the 2D and 3D keypoint detection network performance to the results of 2D and 3D segmentation networks and human intra-reader variation. For experiments with more than two sets of data samples, the Kruskal–Wallis test was applied first; if the differences were significant across the sets, comparisons were then conducted between pairs of samples using the Wilcoxon signed-rank test. Multiple comparisons were accounted for by using Bonferroni correction. A *p* < 0.001 was considered significant.

## Results

### Needle localization on SS-CB MR images

**Fig. 4:**
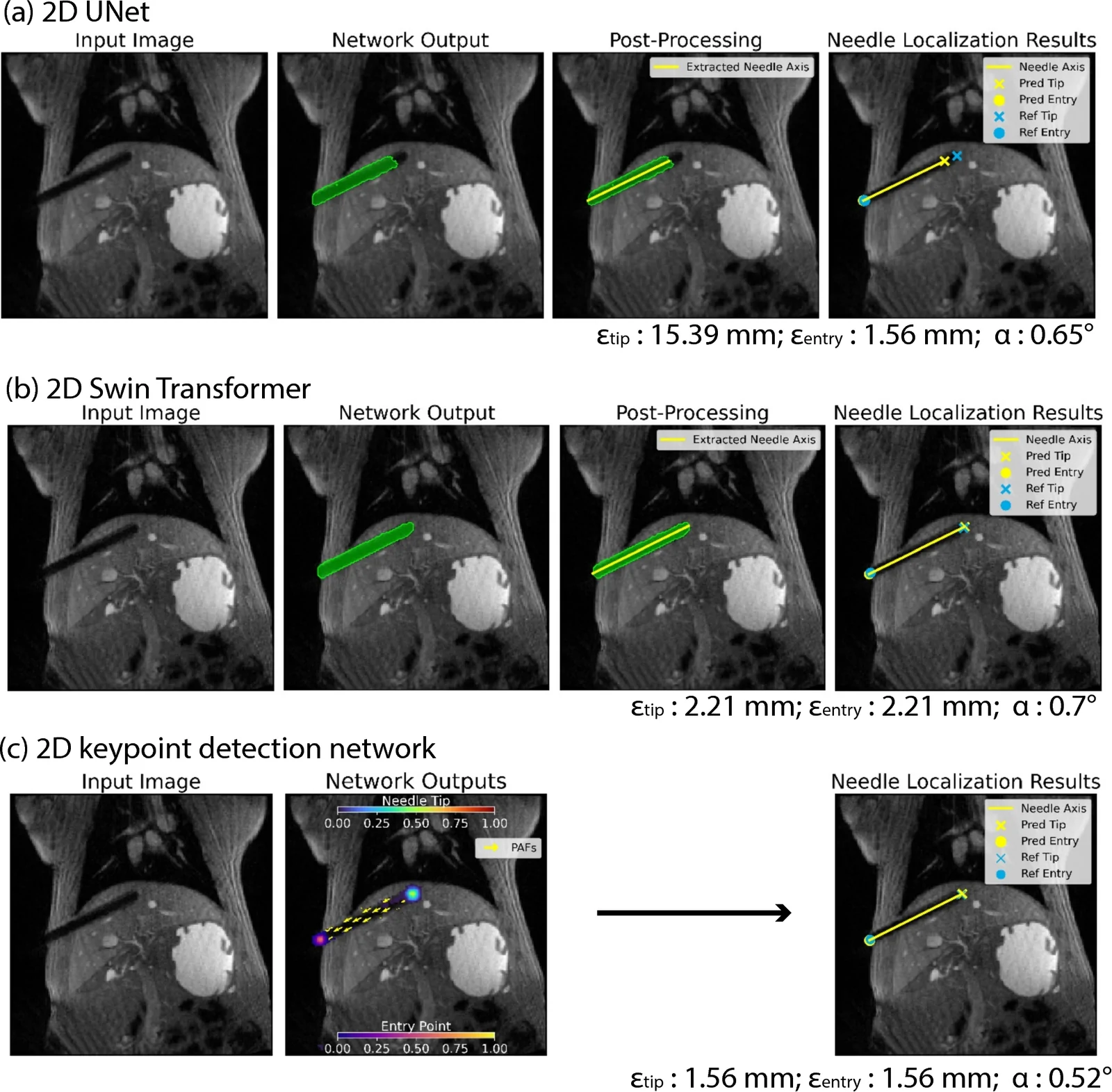
Example of needle localization on a single-slice controlled-breathing (SS-CB) MR image using **a** 2D UNet, **b** 2D Swin Transformer, and **c** 2D keypoint detection network. Reference needle tip and entry point annotations were shown as a blue cross and dot. The prediction results were shown in yellow. The 2D keypoint network directly predicted the locations of the needle tip and entry point without the need for an additional post-processing step of needle axis and coordinate extraction

**Fig. 5:**
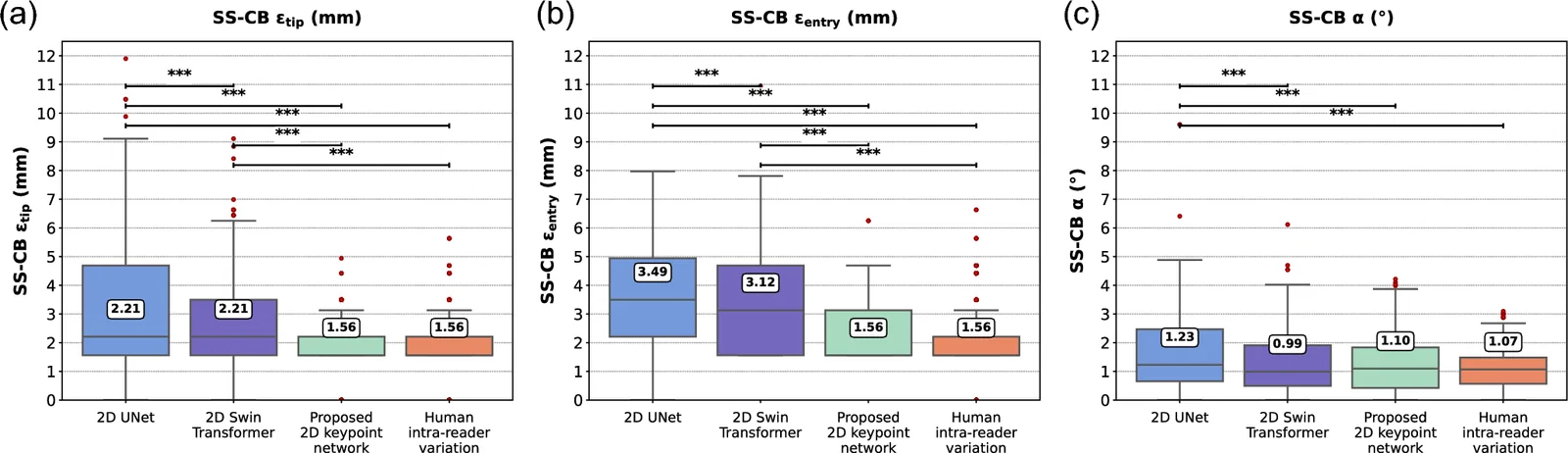
Box plots with swarm plots of **a** needle tip, **b** entry point, and **c** needle axis localization results from the 2D UNet, 2D Swin Transformer, the proposed 2D keypoint detection network, and human intra-reader variation for the single-slice controlled-breathing (SS-CB) independent testing dataset. The numbers shown on the box plots are the medians of the results. In the pair-wise comparisons, *p*-values of the Wilcoxon signed-rank tests are shown on the connecting lines. *** indicates *p* < 0.001

For the proposed 2D keypoint detection network, the average inference time on one NVIDIA RTX A6000 GPU card (48 GB memory) was ~ 10 ms per image, comparable to that of the 2D UNet and 2D Swin Transformer. Figure 4 shows an example of needle localization results for the SS-CB dataset, where the 2D UNet failed in needle tip localization and 2D Swin Transformer exhibited under-segmentation due to motion artifact around the needle tip. Overall, in the independent testing set (SS-CB), the median ${{\boldsymbol{\varepsilon}}}_{{\boldsymbol{t}}{\boldsymbol{i}}{\boldsymbol{p}}}$ and $\boldsymbol{\alpha }$ were 1.56 mm (0% outliers) and 1.10° for the 2D keypoint detection network, which significantly (*p* < 0.001) outperformed the 2D UNet (median ${{\boldsymbol{\varepsilon}}}_{{\boldsymbol{t}}{\boldsymbol{i}}{\boldsymbol{p}}}$ 2.21 mm; 22.86% outliers) and 2D Swin Transformer (2.21 mm; 6.61% outliers) and was comparable to human intra-reader variation (Fig. 5). Online Resource 1 shows needle tracking results on an SS-CB image time series.

### Needle localization on MS-CB MR images

**Fig. 6:**
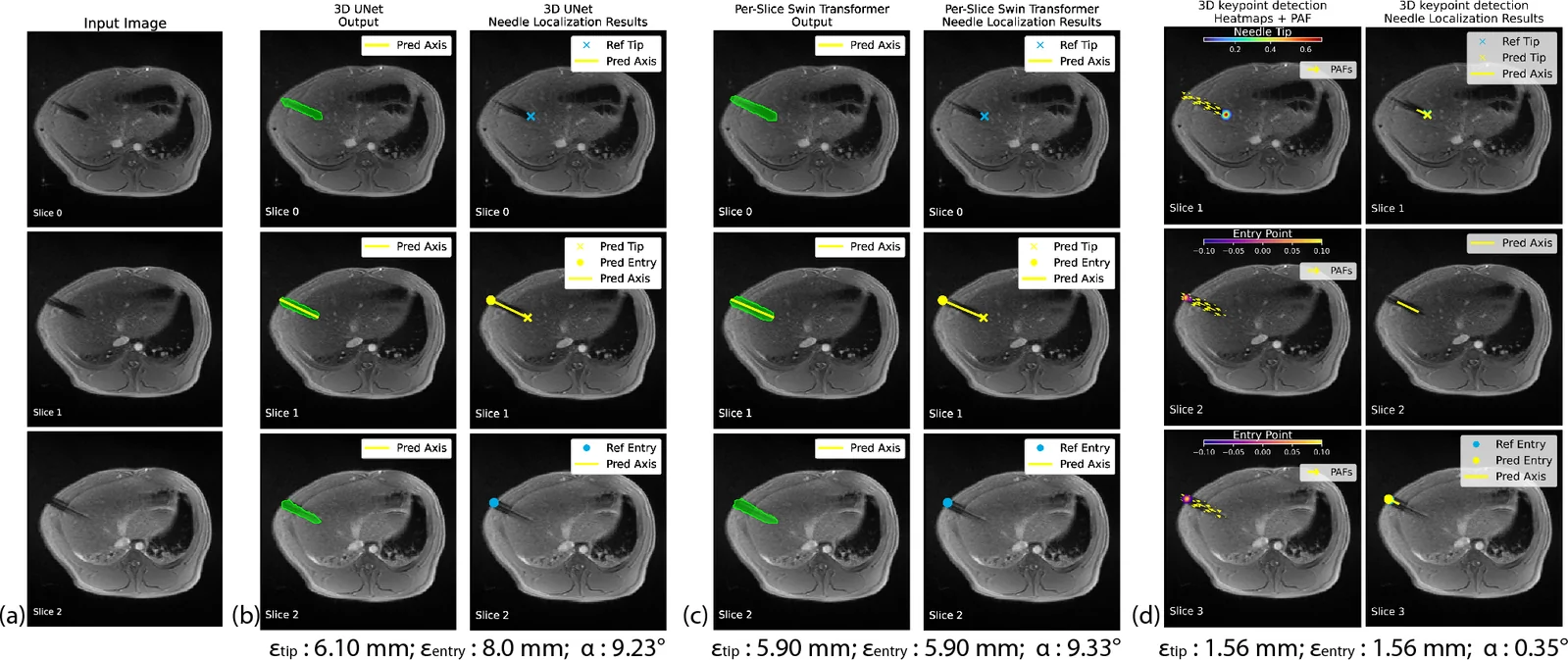
Example of needle localization on a multislice controlled-breathing (MS-CB) MR image set **a** using 3D UNet **b**, Per-slice Swin Transformer **c**, and the 3D keypoint detection network **d**. The needle axis was shown as a disconnected line if the needle axis crossed multiple slices

**Fig. 7:**
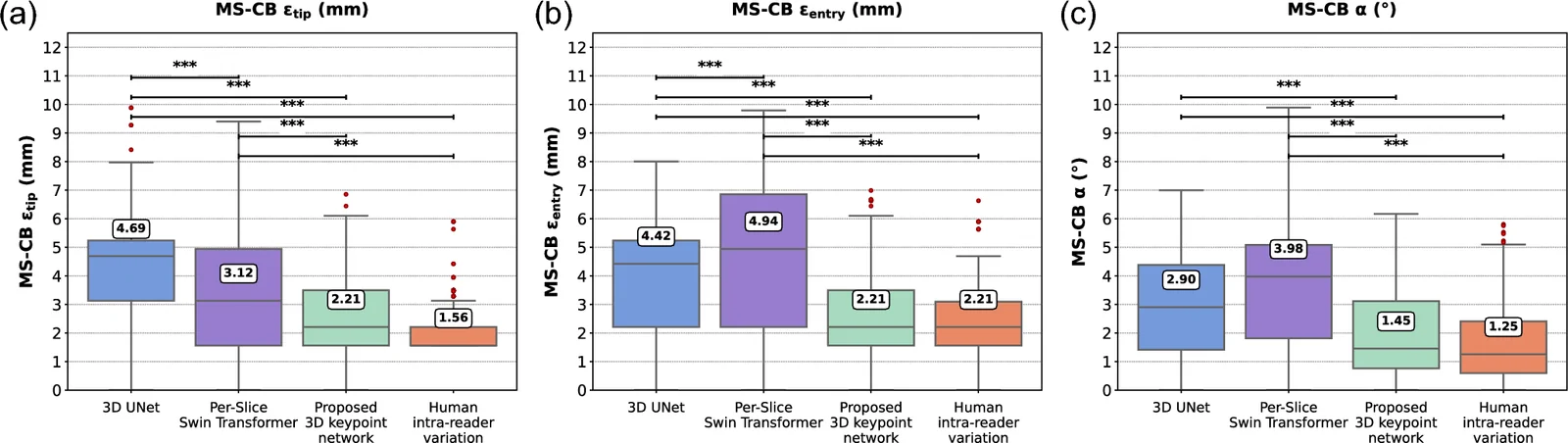
Box plots with swarm plots of **a** needle tip, **b** entry point, and **c** needle axis localization results from the 3D UNet, Per-slice Swin Transformer, and the proposed 3D keypoint network, and human intra-reader variation on the multislice controlled-breathing (MS-CB) independent testing dataset. The numbers shown on the box plots are the medians of the results. In the pair-wise comparisons, *p*-values of the Wilcoxon signed-rank tests are shown on the connecting lines. *** indicates *p* < 0.001

For the proposed 3D keypoint detection network, the inference time was 30 ms per image set on an NVIDIA RTX A6000 GPU and was similar to that for the 3D UNet and the total processing time of the per-slice Swin Transformer. Examples of needle localization for the MS-CB datasets are shown in Fig. 6. These examples highlight that ambiguities in the segmentation masks from 3D UNet can lead to incorrect predictions in the slice index during post-processing of the masks. In the independent testing set (MS-CB), the median ${{\boldsymbol{\varepsilon}}}_{{\boldsymbol{t}}{\boldsymbol{i}}{\boldsymbol{p}}}$ and $\boldsymbol{\alpha }$ were 2.21 mm (7.95% outliers) and 1.45° for the 3D keypoint detection network, which also significantly (*p* < 0.001) outperformed the 3D UNet (median ${{\boldsymbol{\varepsilon}}}_{{\boldsymbol{t}}{\boldsymbol{i}}{\boldsymbol{p}}}$ 4.69 mm; 38.24% outliers) and per-slice Swin Transformer (3.12 mm; 20.29% outliers) and achieved comparable performance as human intra-reader variation (Fig. 7). Online Resource 2 shows needle tracking results on an MS-CB image set series over multiple time frames.

### Ablation experiments

**Fig. 8:**
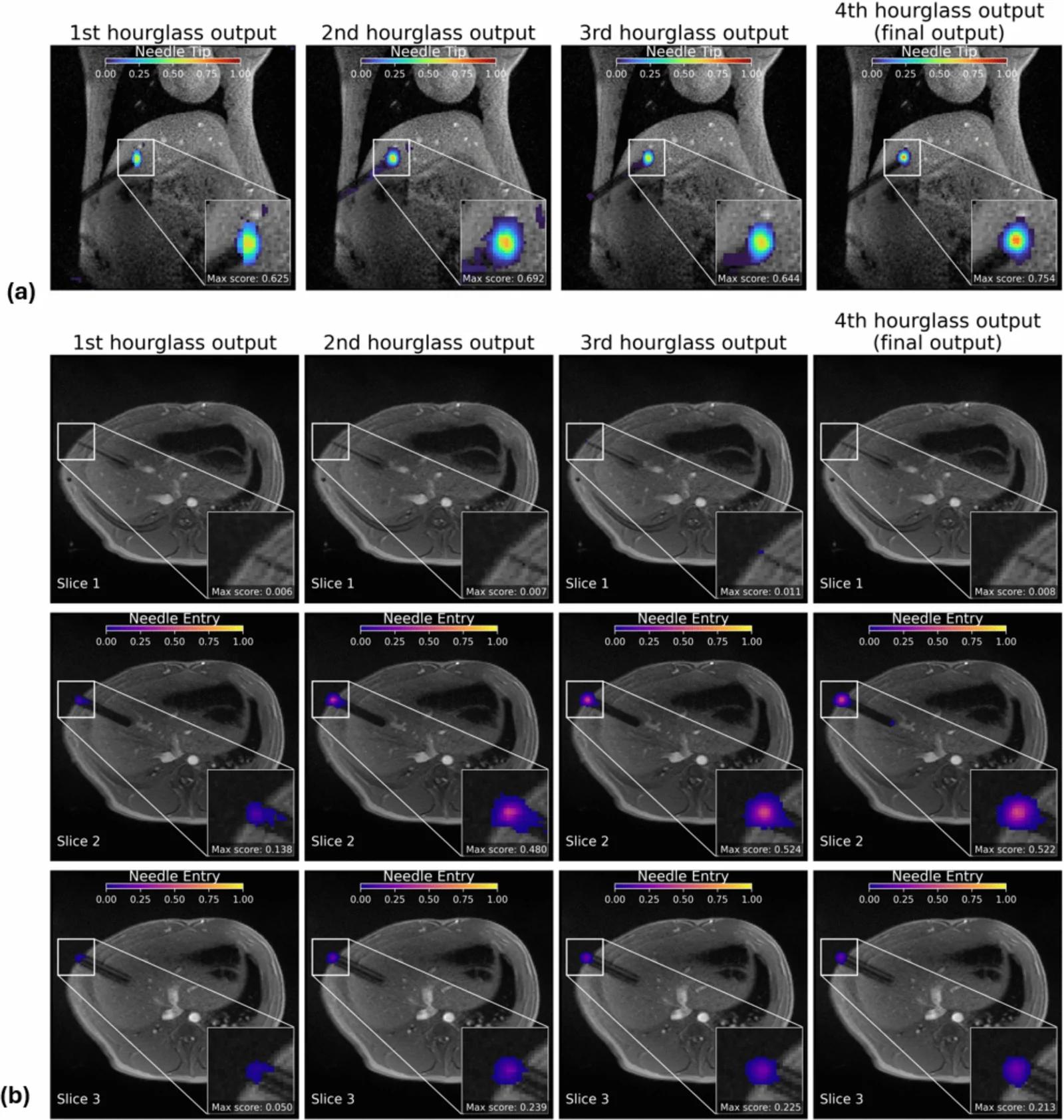
The predicted heatmaps from the intermediate (stacks 1–3) and the final hourglass module (stack 4) for the single-slice controlled-breathing (SS-CB) image (**a**) and the multislice controlled-breathing (MS-CB) image (**b**). Each hourglass module iteratively refined the predicted heatmaps to improve localization accuracy. For visualization, **a** is only showing the heatmap of the needle tip and **b** is only showing the heatmap of the needle entry point

We performed ablation experiments to study the effects of the number of stacked hourglass modules and the strategy of multi-task learning with keypoint and PAF prediction. Figure 8 presents a representative example where the stacked hourglass architecture incrementally improved heatmap accuracy and geometric consistency with each additional module. For 2D keypoint detection, median ${{\boldsymbol{\varepsilon}}}_{{\boldsymbol{t}}{\boldsymbol{i}}{\boldsymbol{p}}}$ improved from 2.21 mm (15.44% outliers) with 1 module to 1.56 mm (0% outliers) with 4 modules. For 3D models, median ${{\boldsymbol{\varepsilon}}}_{{\boldsymbol{t}}{\boldsymbol{i}}{\boldsymbol{p}}}$ improved from 2.21 mm (24.12% outliers) with 1 module to 2.21 mm (7.95% outliers) with 4 modules.

**Fig. 9:**
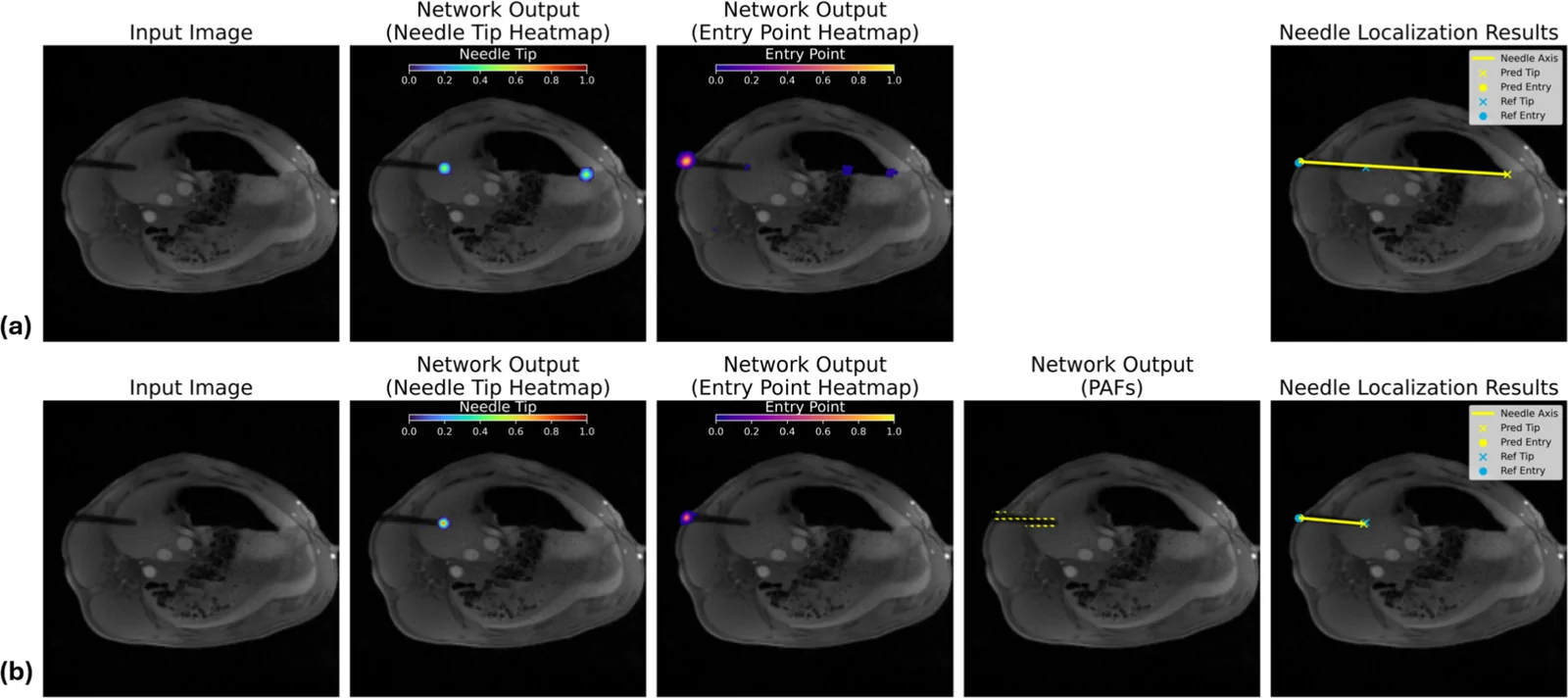
Needle localization results on a single-slice breath-holding (SS-BH) MR image using a 2D keypoint detection network model trained without the part affinity fields (PAFs) prediction task **a**, and a model trained with the PAFs prediction task **b**. The network without PAFs prediction had a false prediction of the needle tip location. The heatmaps for needle tips and entry points are shown separately

We also trained 2D keypoint detection models with and without the PAF prediction task. Figure 9 shows an example where the model without PAFs had false-positive needle tip localization due to the lack of connectivity representation learnt from the PAF. Model training with PAF prediction reduced ${{\boldsymbol{\varepsilon}}}_{{\boldsymbol{t}}{\boldsymbol{i}}{\boldsymbol{p}}}$ outliers for both 2D models (12.74% to 0%) and 3D models (13.35% to 7.95%).

## Discussion

In this study, we developed and evaluated 2D and 3D keypoint detection models for needle localization on 2D single-slice and 2D multislice intra-procedural MR images acquired during breath-holding and controlled-breathing in pre-clinical pig models. The proposed 2D keypoint detection network achieved a median ${{\boldsymbol{\varepsilon}}}_{{\boldsymbol{t}}{\boldsymbol{i}}{\boldsymbol{p}}}$ of 1.56 mm (1 pixel) (0% outliers) and $\boldsymbol{\alpha }$ of 1.10° for the independent testing set of SS-CB images. The proposed 3D keypoint detection network achieved a median ${{\boldsymbol{\varepsilon}}}_{{\boldsymbol{t}}{\boldsymbol{i}}{\boldsymbol{p}}}$ of 2.21 mm (~ 1.5 pixels) (7.95% outliers) and $\boldsymbol{\alpha }$ of 1.45° for the independent testing set of MS-CB images. Both 2D and 3D proposed networks showed performance comparable to human intra-variation.

For comparison, we trained and evaluated U-Net and Swin Transformer models as representative segmentation baselines commonly used in segmentation model evaluation [13, 32, 33]. These models localized the needle using segmentation masks rather than heatmaps [34]. Both segmentation models exhibited a higher number of needle tip localization outliers in 2D and 3D evaluations, which can be attributed to the fact that segmentation architectures [35] and the loss functions (e.g., Dice and Hausdorff distance) are optimized for dense mask prediction rather than precise keypoint localization [36]. As illustrated in Fig. 4, even segmentation results achieving high Dice scores can still exhibit major errors in needle tip localization. In contrast, the keypoint detection networks utilized weighted MSE loss to directly assess the predicted heatmap of the keypoints, which improved the needle tip and entry point localization accuracy. Moreover, on the multislice datasets, ambiguity in defining segmentation masks may lead to needle tip or entry point slice index prediction error (Fig. 6). The keypoint detection model directly predicts the heatmaps of the needle tip and entry point and therefore achieved more robust performance on the multislice dataset compared to the results of the segmentation networks.

Another distinctive advantage of the proposed keypoint detection networks is the dramatically shorter annotation time, especially for 3D or 2D multislice images. Whereas segmentation requires careful slice-by-slice tracing to ensure the integrity, keypoint annotation only requires marking two points [10]. With the efficient annotation process, the proposed keypoint detection models could be adapted to localize other interventional devices, such as ablation needles and catheters, especially in cases involving complex device structures or where extracting device coordinates from segmentation masks is challenging. Furthermore, the modular stacked hourglass architecture enables application-specific optimization by adjusting the number of hourglass modules to balance inference speed and keypoint localization accuracy.

There were limitations to this study. Firstly, our current method extracted needle keypoint coordinates from heatmaps with respect to the voxel grid of the image, which had in-plane resolution of 1.56 mm/pixel and slice thickness of 5 mm. This constrained keypoint localization accuracy to the resolution of the voxel grid. Future work will explore alternative coordinate extraction strategies from the heatmaps, including image interpolation and soft-argmax heatmap regression [37] to achieve sub-voxel accuracy and use multislice images with reduced slice thickness to improve needle localization accuracy on multislice images. Secondly, this study used pre-clinical datasets acquired during controlled-breathing and did not include free-breathing datasets. However, controlled-breathing is relevant for clinical interventions [3, 24] and provides a representative level of breathing pattern variability. Nevertheless, future work will include larger datasets with diverse breathing conditions to further improve model performance. Thirdly, the proposed keypoint detection networks primarily addressed needle localization during the insertion stage, with the assumption that 2D imaging plane prescription has already been performed along the planned needle trajectory during the planning stage. Using this workflow, we only studied the 3-parallel-slice images as input for the 3D keypoint detection model to account for minor angular deviations that can happen during the insertion stage. Future work can extend the proposed 3D model to multislice inputs with more slices and 3D volumetric inputs for needle localization during the planning stage. Finally, our models were trained/tested with a limited internal dataset. Further research is needed to investigate our models in larger datasets and perform external validation.

## Conclusion

In this work, we developed 2D and 3D keypoint detection models that enable rapid and accurate needle localization in single-slice and multislice 2D intra-procedural liver MRI. The proposed keypoint detection models directly predict the heatmaps of the needle tip and entry point, which leads to a more efficient annotation process and more robust performance in needle localization compared to the segmentation-based results from UNet and Swin Transformer, showing the potential to enhance MRI-guided liver interventions.

## Supplementary Information

Below is the link to the electronic supplementary material.

Supplementary file1 (EPS 16039 KB): Examples of synthetic needle features compared with actual acquired in vivo MRI. (a) The needle feature on actual acquired in vivo multislice 2D golden-angle radial GRE images. (b) The synthetic needle features generated with the same imaging parameters and needle positions. (c) The synthetic needle overlaid on 3-parallel-slice images extracted from 3D gradient-echo MRI collected during the pig experiments (TR/TE: 3.91/1.23 ms, resolution: 1.35x1.35 mm2, slice thickness: 1.5 mm, 120 slices). Images were zoomed in for display.

Supplementary file2 (MP4 62214 KB): Video of the needle tracking result of the proposed 2D keypoint detection network for an SS-CB image time series. The video shows that the keypoint network successfully tracked the needle movement induced by the breathing motion. The frame index and the timestamp are labeled on the upper left of the images.

Supplementary file3 (MP4 32272 KB): Video of the needle tracking result of the proposed 3D keypoint detection network for an MS-CB image time series. The video shows that the keypoint network successfully tracked the needle retraction movement and the movement induced by the breathing motion. The frame index and the timestamp are labeled on the upper left of the images.
